# WHO 2024 Hepatitis B Guidelines and Treatment-Eligible Rate Among Treatment-Naive Patients

**DOI:** 10.1001/jamanetworkopen.2024.35777

**Published:** 2024-09-27

**Authors:** Jian Wang, Shaoqiu Zhang, Chuanwu Zhu, Chao Wu, Rui Huang

**Affiliations:** 1 Department of Infectious Diseases, Nanjing Drum Tower Hospital, Affiliated Hospital of Medical School, Nanjing University, Nanjing, Jiangsu, China; 2 Institute of Viruses and Infectious Diseases, Nanjing University, Nanjing, Jiangsu, China; 3 Department of Infectious Diseases, The Affiliated Infectious Diseases Hospital of Soochow University, Suzhou, Jiangsu, China

## Abstract

This cross-sectional study compares rates of hepatitis B treatment eligibility among treatment-naive patients according to the World Health Organization (WHO) 2024 guidelines with eligibility rates across other international guidelines.

## Introduction

Current antiviral medications are effective in suppressing hepatitis B virus (HBV) replication and improving patient prognosis.^1,2,3,4^ However, international HBV guidelines propose different criteria for antiviral treatment.^1,2,3,4^ Only 3% of patients received treatment globally in 2022.^4^ To improve the uptake of antiviral therapy, simplifying and expanding the eligibility for treatment is the future direction of HBV management.^4^ Recently, the World Health Organization (WHO) updated HBV guidelines, with expanded treatment eligibility being a key priority area.^4,5^ However, there is limited information on the differences in treatment eligibility rates among international hepatitis B guidelines. We compared treatment-eligible rates across Asian Pacific Association for the Study of the Liver (APASL) 2015, American Association for the Study of Liver Diseases (AASLD) 2018, European Association for the Study of the Liver (EASL) 2017, and WHO 2024 guidelines.

## Methods

This retrospective cross-sectional study included 12 217 treatment-naive patients with chronic HBV from 2015 to 2023 across 3 medical centers. Treatment criteria of international guidelines used in this study are showed in the eTable in Supplement 1. Significant liver fibrosis was identified by aspartate aminotransferase-to-platelet ratio index (≥1.5), fibrosis index based on 4 factors (≥3.25), liver stiffness measurement (≥8.0 kPa), or liver biopsy (liver histology ≥stage 2 fibrosis). This study was approved by the Institutional Review Board of Nanjing Drum Tower Hospital. Due to the study’s retrospective design, informed consent was waived by the ethics committee of Nanjing Drum Tower Hospital. This study adhered to the ethical guidelines of the Declaration of Helsinki and followed the STROBE reporting guideline for cross-sectional studies.

## Results

Due to insufficient clinical data, 2175 patients were excluded from the analysis. Among 10 042 patients included (median [IQR] age, 37.0 [30.0-48.0] years; 61.5% male), median (IQR) levels of alanine aminotransferase and HBV DNA were 32.1 (20.3-64.5) U/L and 3.2 (2.7-6.2) log_10_IU/mL, respectively, and 30.1% of patients were hepatitis B e antigen (HBeAg) positive (Table). A total of 2318 patients (23.1%), 2773 patients (27.6%), and 2841 patients (28.3%) were treatment eligible according to APASL 2015, AASLD 2018, and EASL 2017 guidelines, respectively, while 6338 patients (63.1%) met the treatment criteria of WHO 2024 guidelines, which was significantly higher than for the other guidelines (*P* < .001) (Figure, A). More patients who were HBeAg positive than negative were treatment eligible in different guidelines, with rates as high as 88.7% for WHO 2024, followed by 65.1% for EASL 2017, 48.0% for AASLD 2018, and 46.9% for APASL 2015 guidelines (*P* < .001) compared with proportions of 52.1%, 12.5%, 17.1%, and 12.9%, respectively, in patients who were HBeAg negative (*P* < .001) (Figure, B). The proportion of treatment-eligible patients for WHO 2024 guidelines was 67.0% in males, which was significantly higher than that for EASL 2017 (31.1%), AASLD 2018 (29.8%), and APASL 2015 (26.4%) guidelines (*P* < .001). A similar trend was observed in females (Figure, C). Older patients had a higher treatment-eligible rate (Figure, D). The numbers of patients who met option 1, 2, and 3 of WHO 2024 guidelines were 2831 patients (28.2%), 3996 patients (39.8%), and 2631 patients (26.2%), respectively (Figure, E).

**Table.** Study Population Characteristics

Characteristic	Overall patients, No. (%) (N = 10 042)
Age, median (IQR), y	37.0 (30.0-48.0)
Sex	
Male	6175 (61.5)
Female	3867 (38.5)
Family history of HCC or cirrhosis	180 (1.8)
Liver cirrhosis	896 (8.9)
Liver steatosis	1997 (19.9)
Diabetes	621 (6.18)
Laboratory values, median (IQR)	
Platelet count, ×10^3^/μL	190.0 (149.0-229.0)
Total bilirubin, mg/dL	0.8 (0.6-1.1)
ALT, U/L	32.1 (20.3-64.5)
AST, U/L	26.2 (20.6-42.9)
GGT, U/L	23.2 (16.0-42.1)
Albumin, g/L	44.7 (42.7-46.4)
HBeAg positive	3018 (30.1)
HBV DNA (log_10_ IU/mL)	3.2 (2.7-6.2)
APRI score (n = 7045)	
Median (IQR)	0.35 (0.25-0.70)
≥1.5	933 (13.2)
≥2.0	741 (10.5)
FIB-4 score (n = 7044)	
Median (IQR)	1.00 (0.65-1.72)
≥3.25	769 (10.9)
≥6.5	309 (4.4)
LSM, kPa (n = 1165)	
Median (IQR)	6.2 (4.8-7.8)
≥8.0	270 (23.2)
≥11.0	108 (9.3)
Liver biopsy (n = 227)	
≥S2	100 (44.1)
≥G2	115 (50.7)

Abbreviations: ALT, alanine aminotransferase; APRI, aspartate aminotransferase to platelet ratio index; AST, aspartate aminotransferase; FIB-4, fibrosis index based on 4 factors; HBeAg, hepatitis B e antigen; HBV, hepatitis B virus; HCC, hepatocellular carcinoma; GGT, γ-glutamyltransferase; LSM, liver stiffness measurement.

SI conversion factors: To convert ALT, AST, and GGT to microkatals per liter, multiply by 0.0167; albumin to grams per liter, multiply by 10; platelet count to ×10^9^ per liter, multiply by 1.0; and total bilirubin to micromoles per liter, multiply by 17.104.

**Figure.**
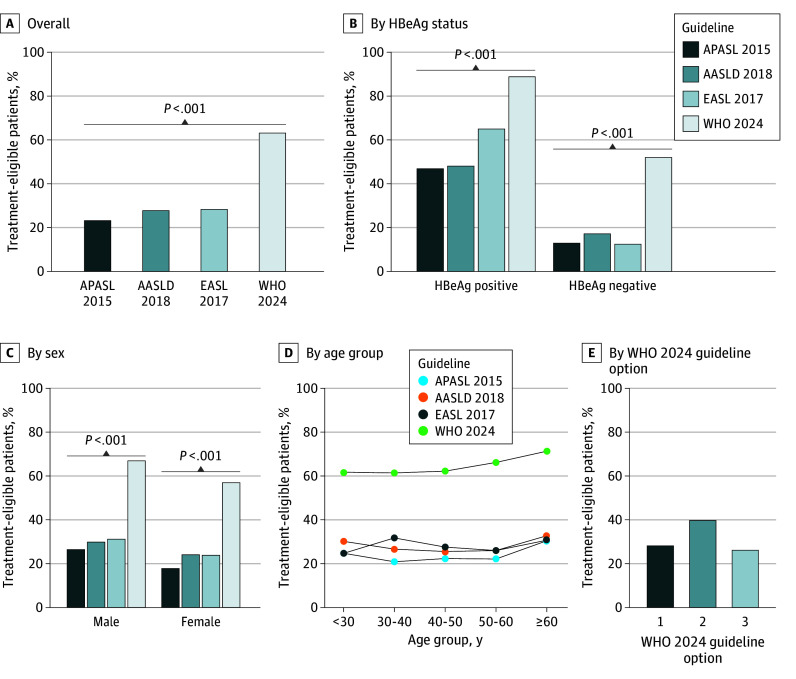
Treatment Eligibility by International Guideline Eligibility is shown among overall patients (A) and by subgroups of hepatitis B e antigen (HBeAg) status (B), sex (C), age (D), and proportions of patients meeting each component of the World Health Organization (WHO) 2024 guidelines (E). Option 1 was evidence of significant fibrosis based on an aspartate aminotransferase-to-platelet ratio index (APRI) greater than 0.5 or transient elastography value greater than 7 kPa or evidence of cirrhosis (APRI >1.0 or transient elastography value >12.5 kPa). Option 2 was hepatitis B virus DNA level greater than 2000 IU/mL and alanine aminotransferase level greater than 1 × the upper limit of normal. Option 3 was the presence of coinfections (HIV, hepatitis D virus, or hepatitis C virus), a family history of liver cancer or cirrhosis, immune suppression, comorbidities (diabetes or metabolic dysfunction–associated steatotic liver disease), or extrahepatic manifestations. AASLD indicates American Association for the Study of Liver Diseases; APASL, Asian Pacific Association for the Study of the Liver; EASL, European Association for the Study of the Liver.

## Discussion

In this cross-sectional study, we found that the WHO 2024 guidelines had the highest treatment-eligible rate compared with other international guidelines. Although the treatment-eligible rate increased significantly according to the WHO 2024 guidelines, efforts to increase treatment uptake and linkage to care are equally important. A study limitation was that all patients were retrospectively included from liver centers or hospitals in China; therefore, further prospective studies involving racially and ethnically diverse populations and broader regions of the general HBV-infected population are needed to validate our findings.

## Notes

Supplement 1. **eTable.** Antiviral treatment criteria of patients with chronic HBV infection in 4 international guidelines

Supplement 2.
Data Sharing Statement
