# Supplementary material for: WHO 2024 Hepatitis B Guidelines and Treatment-Eligible Rate Among Treatment-Naive Patients
Source: JAMA Netw Open. 2024 Sep 27;7(9):e2435777. doi: 10.1001/jamanetworkopen.2024.35777 (PMC11437376; doi:10.1001/jamanetworkopen.2024.35777)
Supplement: Supplement 1. — eTable. Antiviral treatment criteria of patients with chronic HBV infection in 4 international guidelines [file jamanetwopen-e2435777-s001.pdf]

Supplemental Online Content

Wang J, Zhang S, Zhu C, Wu C, Huang R. WHO 2024 hepatitis B guidelines and treatment-eligible rate among treatment-naive patients with HBV. *JAMA Netw Open*. 2024;7(9):e2435777. doi:10.1001/jamanetworkopen.2024.35777

eTable. Antiviral treatment criteria of patients with chronic HBV infection in 4 international guidelines

This supplemental material has been provided by the authors to give readers additional information about their work.

© 2024 Wang J et al. *JAMA Network Open.*

eTable. Antiviral treatment criteria of patients with chronic HBV infection in four international guidelines.

	HBeAg status	HBV DNA (IU/ml)	ALT	Liver inflammation	Liver fibrosis	Other
AASLD 2018 guidance	+	>20,000	>2 × ULN			
	+	>20,000			≥F2	
	+	>20,000		≥A2		
	-	>2,000	>2 × ULN			
	-	>2,000			≥F2	
	-	>2,000		≥A2		
		≥low level viremia			Compensated cirrhosis	
					Decompensated cirrhosis	
EASL 2017 guidelines		>20,000	>2 × ULN			
		>2,000	>1 × ULN	≥A2		
		>2,000			≥F2	
		Detectable			Cirrhosis	
	+	>20,000				Age >30 years
						Family history of HCC or cirrhosis
						Extrahepatic manifestation
APASL 2015 guidelines	+	>20,000	>2 × ULN			
	+	>20,000			≥F2	
	+	>20,000		≥A2		
	-	>2,000	>2 × ULN			
	-	>2,000			≥F2	
	-	>2,000		≥A2		

© 2024 Wang J et al. *JAMA Network Open.*

		>1 × ULN	≥A2		
		>1 × ULN		≥F2	
	>2,000			Compensated cirrhosis	
	Detectable			Decompensated cirrhosis	
WHO 2024 guidelines	>2,000	>1 × ULN			
				Evidence of significant fibrosis (APRI >0.5 or LSM value >7kPa) or cirrhosis (APRI >1.0 or LSM value >12.5kPa)	
					Presence of co-infections (HIV, HDV, or HCV), a family history of liver cancer or cirrhosis, immune suppression, co-morbidities (diabetes or metabolic dysfunction-associated steatotic liver disease), or extrahepatic manifestations

AASLD: American Association for the Study of Liver Diseases; ALT: alanine aminotransferase; APASL: Asian Pacific Association for the Study of the Liver; EASL: European Association for the Study of the Liver; HBeAg: hepatitis B e antigen; HBV: hepatitis B virus; LSM, liver stiffness measurement; ULN: upper limit of normal; WHO: World Health Organization.

The ULN of ALT: AASLD, 35 U/L for male and 25 U/L for female; EASL, 40 U/L; APASL, 30 U/L for male and 19 U/L for female; WHO, 30 U/L for male and 19 U/L for female.

© 2024 Wang J et al. *JAMA Network Open.*
