# Supplementary material for: WHO 2024 Hepatitis B Guidelines and Treatment-Eligible Rate Among Treatment-Naive Patients
Source: JAMA Netw Open. 2024 Sep 27;7(9):e2435777. doi: 10.1001/jamanetworkopen.2024.35777 (PMC11437376; doi:10.1001/jamanetworkopen.2024.35777)
Supplement: Supplement 2. — Data Sharing Statement [file jamanetwopen-e2435777-s002.pdf]

Data Sharing Statement

Wang. WHO 2024 Hepatitis B Guidelines and Treatment-Eligible Rate Among Treatment-Naive Patients With HBV. *JAMA Netw Open*. Published online September 27, 2024. doi:10.1001/jamanetworkopen.2024.35777

Data
Data available: No

Additional Information
Explanation for why data not available: Due to the privacy protection, the dataset used in this study is available from the corresponding author upon reasonable request.
